# Gel Formation in Protein Amyloid Aggregation: A Physical Mechanism for Cytotoxicity

**DOI:** 10.1371/journal.pone.0094789

**Published:** 2014-04-16

**Authors:** Daniel Woodard, Dylan Bell, David Tipton, Samuel Durrance, Lisa Cole, Bin Li, Shaohua Xu

**Affiliations:** 1 InnoMedic Health Applications, Inc., Kennedy Space Center, Florida, United States of America; 2 Department of Physics and Space Sciences, Florida Institute of Technology, Melbourne, Florida, United States of America; 3 Aerospace Medicine and Occupational Health Branch, Kennedy Space Center, Florida, United States of America; 4 Biological Sciences Department, Florida Institute of Technology, Melbourne, Florida, United States of America; 5 Institute of Human Nutrition, Columbia University, New York, New York, United States of America; University of Akron, United States of America

## Abstract

Amyloid fibers are associated with disease but have little chemical reactivity. We investigated the formation and structure of amyloids to identify potential mechanisms for their pathogenic effects. We incubated lysozyme 20 mg/ml at 55C and pH 2.5 in a glycine-HCl buffer and prepared slides on mica substrates for examination by atomic force microscopy. Structures observed early in the aggregation process included monomers, small colloidal aggregates, and amyloid fibers. Amyloid fibers were observed to further self-assemble by two mechanisms. Two or more fibers may merge together laterally to form a single fiber bundle, usually in the form of a helix. Alternatively, fibers may become bound at points where they cross, ultimately forming an apparently irreversible macromolecular network. As the fibers assemble into a continuous network, the colloidal suspension undergoes a transition from a Newtonian fluid into a viscoelastic gel. Addition of salt did not affect fiber formation but inhibits transition of fibers from linear to helical conformation, and accelerates gel formation. Based on our observations, we considered the effects of gel formation on biological transport. Analysis of network geometry indicates that amyloid gels will have negligible effects on diffusion of small molecules, but they prevent movement of colloidal-sized structures. Consequently gel formation within neurons could completely block movement of transport vesicles in neuronal processes. Forced convection of extracellular fluid is essential for the transport of nutrients and metabolic wastes in the brain. Amyloid gel in the extracellular space can essentially halt this convection because of its low permeability. These effects may provide a physical mechanism for the cytotoxicity of chemically inactive amyloid fibers in neurodegenerative disease.

## Introduction

A wide range of proteins and peptides are capable, under appropriate conditions, of aggregating to form fibers with a unique structure commonly referred to as amyloid. The accumulation of these fibers is a consistent feature of at least a dozen pathological conditions in humans, including both Alzheimer's and Parkinson's diseases. Detailed structures have been modeled or proposed for some amyloid fibers, particularly for those formed from short peptides [1]–[3], however the complete molecular structure for most amyloid fibers has not been determined. A unique feature of amyloid fibers is that protein conformation is radically changed from the native state. Most proteins are predominately alpha-helix. Amyloid fibers, in contrast, characteristically contain a large percentage of an unusual secondary structure called cross-beta-sheet, in which peptide backbones from multiple protein monomers are straightened, arranged in parellel, and joined together by hydrogen bonds [4], [5]. The energy source for the major conformational change that occurs when proteins aggregate remains a matter of active debate. Based on evidence from atomic force microscopy (AFM) and transmission electron microscopy (TEM), Xu proposed that colloidal interactions drive amyloid aggregation, and that aggregation itself drives conformational change [6].

The mechanism of cytotoxicity in amyloid disease also remains uncertain; amyloid fibers are chemically stable and are not known to possess specific biochemical toxicity. Small protein aggregates, or oligomers, are often found with amyloid fibers and may be an intermediate in the aggregation process. These oligomers are more reactive than fibers, and it has been proposed that they play the major role in toxicity [7]. Oligomers, however, consistently appear before fibers, which form much more slowly. If the oligomers are responsible for cytotoxicity, it is difficult to see how a cell could survive long enough to accumulate the large number of intracellular fibers often seen after neuronal death.

Moreover, amyloid aggregation does not end with fiber formation. Molecular and macromolecular fibers of many types, including amyloid, are often seen to self-assemble to form a continuous network. The formation of such a network converts the surrounding suspension from a fluid into a gel, with the striking effect of increasing its viscosity by a factor of as much as 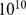 [8]. A wide variety of small and large molecules may act as gelators; despite their different chemical structures, the physical properties of gels appear to be determined largely by pore size, a measure of the expected distance from an arbitrary point in the fluid to the nearest fiber surface [9]. The dramatic physical changes in the intracellular and extracellular environment resulting from gel formation could potentially disrupt transport processes essential for cell survival.

In preparation for an experiment in which amyloid aggregation will be attempted in microgravity aboard the International Space Station, we examined the morphology of amyloid fibers and the kinetics of gel formation under various conditions using the common laboratory model of hen egg white lysozyme. Amyloid aggregation in this model is accelerated as lysozyme becomes unfolded at 55C and peaks at 65C before amorphous aggregation becomes the dominate pathway [10]. Salt concentration has been shown to strongly influence the aggregation of lysozyme [11]–[13]; we therefore examined lysozyme aggregation under a range of salt concentrations. We then considered the potential effects of amyloid gel formation on intra- and extracellular transport.

## Materials and Methods

Buffer was prepared with 10 mM glycine in DI water, titrated to pH 2.5 with HCl. We have found the anion concentration to be critical; to ensure uniformity across samples, the titration was performed in the stock solution prior to the addition of lysozyme; the final HCl concentration was approximately 36 mM. The buffer was prepared with no added salt or with NaCl at concentrations of 30 mM or 150 mM. Lysozyme (BSG, Napa, CA) at a concentration of 20 mg/ml was dissolved in the buffer and 1 ml samples were prepared in capped 1.5 ml microcentrifuge tubes and incubated in an oven at 55C with minimal convection. Limited tests were done with higher concentrations of lysozyme and with agitation at 60 RPM in a Vortemp incubator.

Atomic force microscopy (AFM) slides were prepared from the aggregating protein after up to 30 days of incubation. Samples were applied to slides both undiluted and after serial dilution of up to 1∶10,000 with deionized (DI) water. We found that diluting amyloid fibers by mixing the suspension with water in a conventional vortexer resulted in breakage of virtually all fibers longer than approximately 2
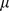
m due to fluid shear. For this reason we performed dilution by the following procedure: For each dilution the tube was gently inverted several times to suspend colloids and 100 microliters was extracted from the center of the tube with a pipette and and added to 900 microliters of DI water, and again mixed by tube inversion. Undiluted and diluted protein samples were preserved at −20C.

It was difficult to draw gels accurately into a standard pipette due to their extreme viscosity. We found by AFM observations that this also resulted in extensive fiber breakage, apparently due to fluid shear as the gel was drawn through the small orifice of the pipette. Consequently, when pipetting fibers and/or gels, the tips of the pipettes were first cut off to enlarge the orifice diameter to 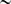
1 mm.

To prepare AFM slides, 10
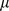
l of 0.01N NaOH was applied to freshly peeled mica to precharge the substrate. After two minutes 10
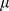
l of the sample was applied to the substrate at the same location. After an additional 10 minutes the sample was gently rinsed with 2 ml of DI water by allowing the water to flow slowly over the substrate in a Petri dish to remove salts and unbound proteins. The water was drained and any droplets adhering to the substrate were removed with a tangentially applied jet of nitrogen gas. The slide was dried for at least 2 hr at 55C.

Samples were imaged with a Molecular Imaging Picoscan Plus AFM in contact mode, using standard long thin-leg Veeco DNP silicon nitride cantilevers. Vertical and horizontal measurement was calibrated and convolution was measured with 20 nm and 200 nm vertical step calibrators. AFM tip radius was typically 10 nm and tips with radius greater than 30 nm were replaced. Topographic mode was used to demonstrate three-dimensional structure and for dimensional measurements, while deflection mode was used to demonstrate shape and texture.

In contact-mode AFM the width of structures smaller than the AFM tip can be greatly exaggerated by convolution. A circular fiber of height H, smaller then the radius R of the AFM tip, will have an apparent width W given approximately by W = 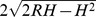 [14]. Consequently we used height rather than width as a measure of the diameter of particles and fibers. Measurement of the length of fibers is not affected because it is much larger than the AFM tip.

Amyloid fibers often display a helical or twisted-ribbon morphology; in the case of primary lysozyme fibers this takes the form of a simple open helical spiral, similar to a stretched spring. When a fiber is bound horizontally to a substrate and viewed from above by AFM this helix has the appearance of a sinusoidal curve. We examined each fiber in a set of images to see if it consisted of at least two consecutive sinusoidal cycles of equal period and amplitude; if it did so it was considered helical. A fiber of comparable length with no sinusoidal curves was considered linear.

## Results

Fiber formation was first seen by AFM after four days of incubation with all three salt concentrations. Three stages of gel formation are visible on gross examination. The first stage is marked by increased fluid viscosity and the appearance of small particles of soft gel in the solution. Visible gel particles are typically 1–3 mm in size and are visible when the tube is held against a lighted background. Smaller particles may be present but would be difficult to see, while larger ones tend to settle to the bottom of the tube. The second stage is marked by conversion of the solution into a clear soft gel which meets the tube inversion criterion [15] but visibly changes in shape on inversion and will eventually fracture on repeated inversion. The third stage demonstrates conversion to a harder gel which does not change shape on inversion and, after about 30 days, develops a faint yellow color.

With the standard preparation of lysozyme without added NaCl, gel particles were seen at day 11 and soft gel formation within 18 days. Hard gel formation was not seen. The addition of NaCl at either 30 mM or 150 mM accelerated gel formation, with increased viscosity and gel particles at day 8, soft gel at day 11, and hard gel at day 22.

Gel formation can be accelerated by increasing lysozyme concentration or by agitation. Lysozyme 40 mg/ml incubated in buffer with 150 mM NaCl resulted in soft gel formation at 8 days. Lysozyme at 20 mg/ml in buffer with 150 mM NaCl, agitated at 60 RPM in a Vortemp incubator, consistently reached the hard gel state within three days.

**Figure 1:**
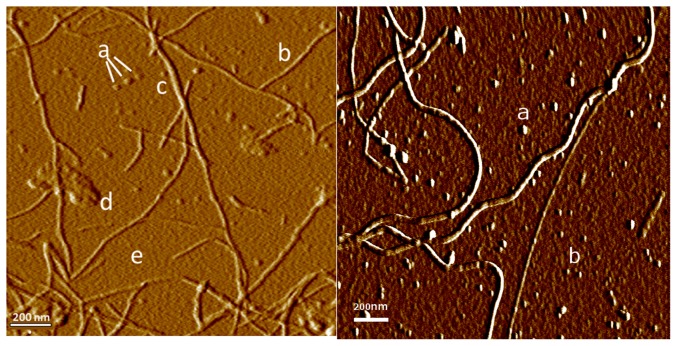
Components of aggregating lysozyme. Left: AFM image showing various structures seen in aggregating lysozyme on mica substrate: (a) colloidal spheres, (b) primary fibers, (c) compound fibers, (d) amorphous aggregates, (e) a continuous layer of protein monomers bound to substrate. Right: After 1∶1000 dilution the continuous protein layer is no longer present. Colloidal spheres are still seen (a), as are numerous discrete particles (b) with a height of 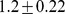
nm (n = 20), probably representing individual lysozyme monomers.

To understand the self-assembly process we examined aggregating lysozyme at various stages using AFM. Early in the process the most common aggregates are colloidal spheres and fibers (Figure 1). Lysozyme fibers often demonstrate a left-handed helical structure. Amorphous aggregates are also present; some appear to be clusters of colloidal spheres. Protein monomers form a confluent layer at high concentrations, in which aggregates and fibers may be partially submerged. When the sample is diluted 1∶1000 or higher before being applied to the substrate, the confluent protein layer is no longer present and numerous discrete particles are seen, probably representing protein monomers and small colloidal aggregates. Finally, amorphous aggregates up to 100 nm in size are occasionally seen. The smallest fibers are typically 1.4–1.6 nm in height; larger fiber bundles up to 8 nm in diameter are also seen.

### Evolution of Fiber Morphology

Under TEM or AFM many gel-forming fibers that have been incubated for an extended period demonstrate a helical appearance. Terech [8] provides a conceptual mechanism for the development of this helicity; initially the fiber is a chain of discoid organic molecules loosely bound by colloidal forces, without specific orientation. Consequently the newly formed fiber has no long-range helical structure and appears linear. With time the monomers rotate into a minimum energy orientation in which each monomer is bound to those adjacent to it by multiple hydrogen bonds. At this stage there is often a structure-dependant angular offset in rotation and tilt between each monomer and the next, resulting in the overall helical shape.

**Figure 2:**
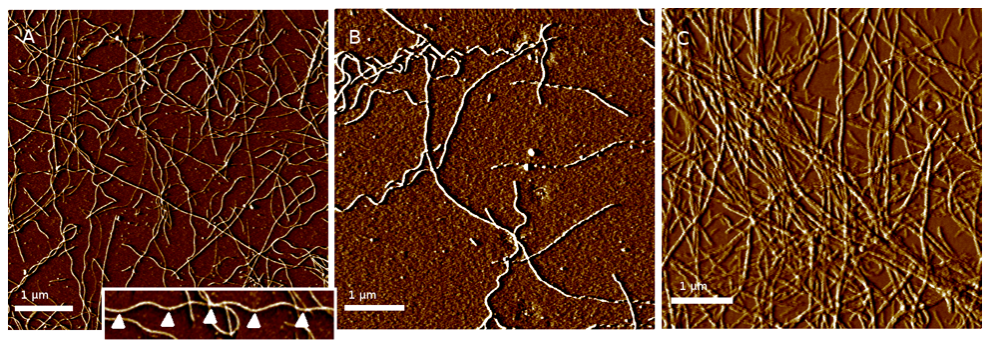
Transition to helical structure is inhibited by high salt concentration. Lysozyme aggregating in buffer with no salt (left) or 30 mM NaCl (center) demonstrate transformation of virtually all fibers to helical structure after incubation for 11 days. The helical curve of the lysozyme fiber bound to the substrate typically appears from above as a sinusoid (left, inset) although other patterns such as twisted ribbons are occasionally seen. The triangular symbols show the length of one turn of the helix is approximately.4
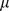
m. Fibers forming in buffer with 150 mM NaCl (right) demonstrate virtually no helical structure even after 31 days.

**Figure 3:**
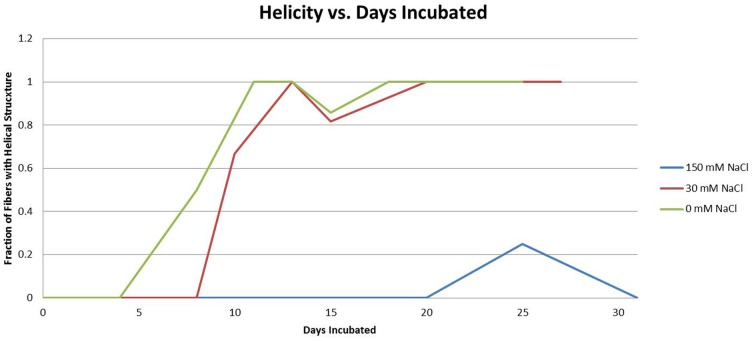
Effect of time and salt concentration on fiber helicity. Newly formed fibers are linear at all salt concentrations; at zero and 30 mM NaCl the fraction of fibers displaying helical morphology increases consistently with time until all fibers are helical. This change occurs more rapidly at lower salt concentrations. At 150 nM NaCl essentially all fibers remain linear.

Our observations were consistent with this model, however the transition to helical morphology in lysozyme was markedly affected by salt concentration (Figure 2). In all buffers, lysozyme fibers were linear in appearance when initially observed. With no added salt, helical fibers first appeared at day 5 and by day 11 essentially all fibers were helical. With the addition of 30 mM NaCl, helical fibers first appeared on day 8 and all fibers were helical by day 13. At 150 mM NaCl the transformation was essentially halted. A few helical fibers were seen late in incubation (day 25), but even at day 31 most samples showed no helical fibers (Figure 3).

### Assembly of Fiber Bundles

**Figure 4:**
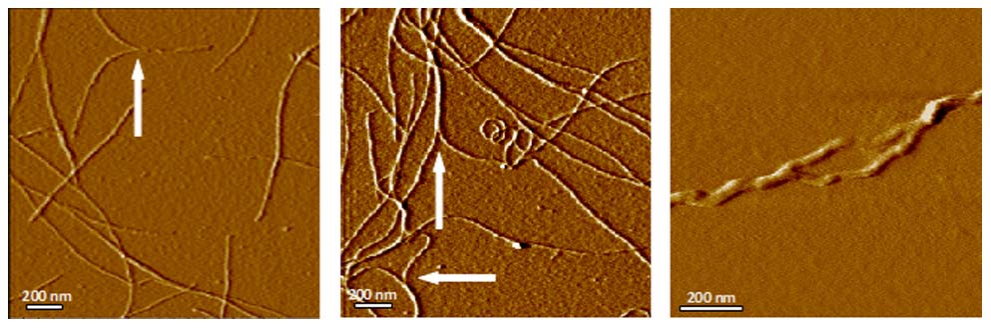
Fiber bundle formation. Lysozyme fibers aggregate by lateral association to form bundles. Left, center: Arrows identify points at which two fibers join to form a bundle. Appearance of the bundle suggests the fibers wrap around each other in a spiral fashion, a process described by Terech [8] as helix formation. This requires the fibers be free to rotate. Right: Lysozyme fibers in a bundle may remain separated over a short distance and then rejoin, suggesting fiber helices must be aligned for bundling to occur.

Examination of gels reveals fibers of several distinct diameters suggesting that primary fibers of a uniform diameter may merge one or more times to form fiber bundles. A single fiber is occasionally seen to divide into two fibers (Figure 4). Is this a single fiber branching into two during growth, or two separately formed fibers in the process of merging to form a larger fiber? Merging of existing fibers seems more probable as the two fibers approaching the junction are invariably smaller, with about half the total cross-sectional area of the fiber leaving the junction. The larger fiber often demonstrates helical structure with the appearance of two fibers twisted together. This is typical of helix formation, a form of fiber self-assembly commonly seen in macromolecular networks [8]. Merging of helical fibers appears to require that they be free to rotate so that the helices of the two fibers can wrap around each other. We occasionally see a single helical fiber that appears to divide into two smaller fibers over a short segment of its length, which then rejoin (Figure 4, right panel). This suggests that merging of the fibers into a bundle was inhibited at this point, perhaps by misalignment of the helices of the merging fibers.

### Assembly of Fiber Networks

On day 4 fibers were seen on AFM, however they were not linked together and the mean distance between fibers on the substrate was 1027 nm, SD = 553 nm (n = 37). No gross gel-like behavior was seen. When gel was present by the tube inversion criterion [15] the mean distance between fibers was 96.5 nm, SD = 38.43 (n = 30), and fibers were linked into a continuous network. This is consistent with Terech's model of the transition from a Newtonian fluid stage to a viscoelastic gel [8].

As incubation was continued past 30 days, the gel changed from an initial colorless appearance to a translucent yellow and appeared stiffer and stronger in consistency. This suggests that chemical changes, possibly oxidation and cross-linking, continue to occur in the fibers. However, little or no change in fiber density or diameter was seen following the transition from fluid to gel. Moreover, even after prolonged incubation of the gel, colloidal spheres and monomers were still seen on AFM. In contrast, when amyloid aggregation occurs without gel formation, these constituents can be rapidly depleted [6]. This suggests that formation of the gel limits further aggregation.

**Figure 5:**
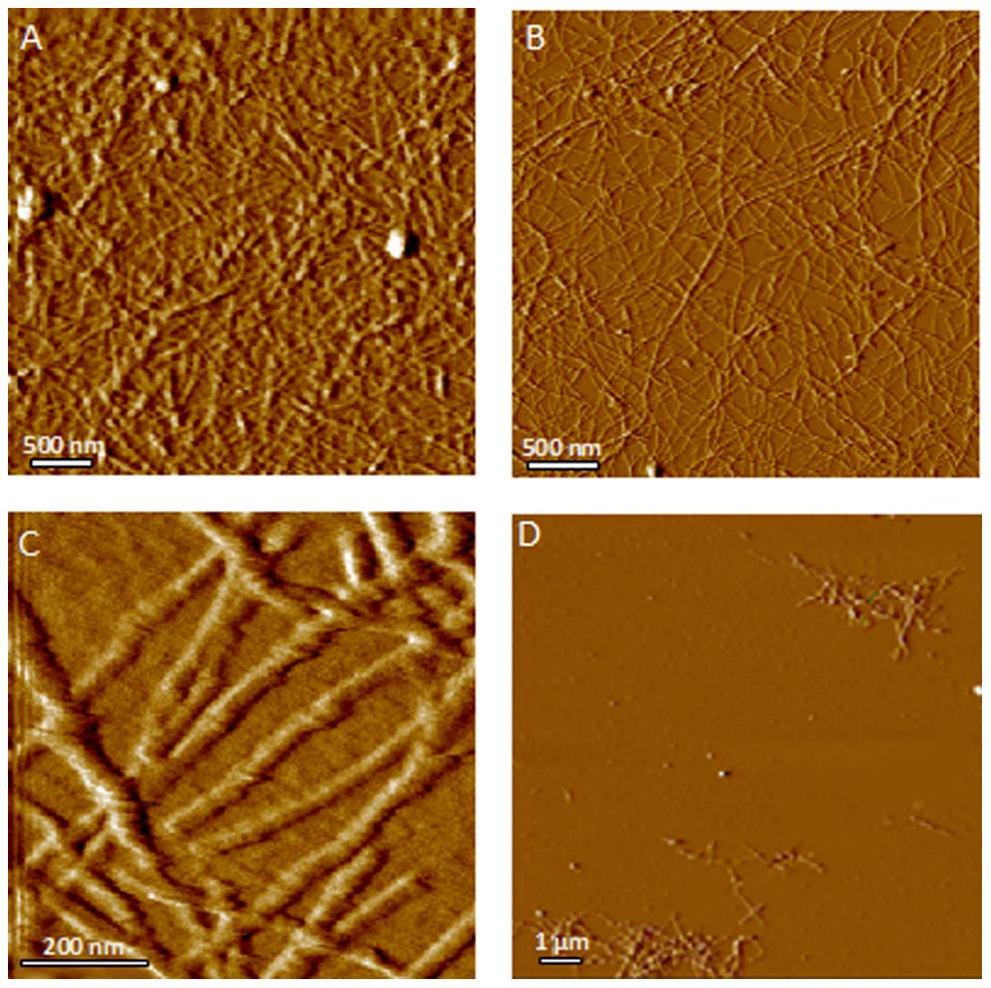
Gel formation in lysozyme. AFM images of lysozyme amyloid fiber network after 30 days incubation. The mixture is a firm gel at this point. (A) Lysozyme gel, undiluted, showing dense network structure. (B) When diluted with water 1∶100 and gently mixed, the gel swells and can be applied to the substrate in a monolayer, showing a range of fiber sizes, bound together at points of contact. (C) Detail of B, showing occasional merging of fibers at points of contact. (D) Gel was diluted in water (1∶10,000) and vortexed ×30 sec. Gel fibers are broken into short fragments but remain joined at points of contact. This indicates development of strong covalent linkages between fibers, typical of an irreversible macromolecular network or IRMAN [8].

Samples of gel applied directly to mica without dilution (Figure 5, panel A) demonstrate a dense, interconnected network of fibers. When lysozyme gels were diluted 1∶100 in DI water and mixed by repeated tube inversion, the gel swelled considerably and a 10
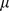
l sample could be applied to mica in a monolayer (Figure 5, panel B, enlarged in panel C). This demonstrates fibers of several diameters; each fiber appears to be uniform in diameter over distances of several microns. Fibers appear to merge where they come into contact at crossing points in the network.

Early in the aggregation process, when the solution is incubated without agitation, the solution appears heterogeneous by AFM. The majority of the slide shows only scattered fibers, however there are occasional clumps of fibers 3–5
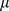
m in size, comparable to the size of physiological neurofibrillary tangles seen in Alzheimer's disease [16]. Grossly, one may see islands of gel 1–3 mm in diameter suspended in the solution. This initial assembly may be due to relatively weak hydrophobic interactions or van der Waals forces at areas of contact between fibers.

As noted, vortexing at normal speed breaks lysozyme fibers into short (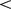
2
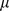
m) segments. When newly formed gels were extracted by pipette, diluted 1∶10,000 in DI water followed by vortexing (30 sec) and applied to mica, short fiber segments were seen on AFM, uniformly dispersed on the substrate. When samples of hard gel incubated 30 days were similarly diluted, vortexed, and applied to a mica substrate AFM again revealed fibers broken into short segments, but they were not uniformly dispersed. Instead they were found in widely scattered clumps, with fiber segments still bound together at points of contact (Figure 5, panel D). This suggests that the bonds between the fibers at points of contact become stronger with time, and after 30 days are comparable in strength to the fibers themselves, so that the fiber may break before the bond at the point of contact. This is consistent, under Teresch's taxonomy, with evolution from a reversible macromolecular network, or REMAN, to an irreversible macromolecular network or IRMAN [8].

## Discussion

The kinetics of amyloid aggregation are highly dependent on incubation conditions. A pH of 2.5 results in partial unfolding of lysozyme, exposing hydrophobic regions and making it more susceptible to aggregation. At pH 2.5, however, lysozyme has a net charge of approximately +16 [17], resulting in coulomb repulsion between lysozyme molecules which could inhibit aggregation.

The presence of salt in the solution can alter protein conformation and affect the colloidal forces between monomers, small aggregates, and fibers which appear critical in amyloid aggregation. If negatively-charged counterions are present they would form a double layer around the positively-charged lysozyme molecule which would be expected to mask these coulomb forces, as described by the theory of Derjaguin, Landau, Verwey and Overbeek (DLVO) [18].

Terech [19], working with aggregation of lithocholate, found that increasing salt concentration led to a change from helical to cylindrical fiber morphology similar to the differences we observed in lysozyme. He ascribed the difference in fiber morphology to shielding of coulomb repulsion between like-charged structures in the fiber, resulting in a change in the conformation of the fiber helix from an open spiral to a closed tube. Increasing salt concentration also led to a striking acceleration in the aggregation of fiber bundles into a gel-forming network. The presence of salt has also been found to accelerate the transition from fluid to gel phase in 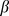-lactoglobulin [20] and in lysozyme/ethanol solutions[12]. Both differences in fiber morphology and shielding of coulomb repulsion between fibers might contribute to the acceleration of gel formation by salt. The differences we saw in aggregation of lysozyme were similar to those seen by Terech in lithocholate, i.e. a visible helical structure appeared only at low salt concentration and gel formation was accelerated in the presence of salt. Whether similar mechanisms are involved is unknown.

**Figure 6:**
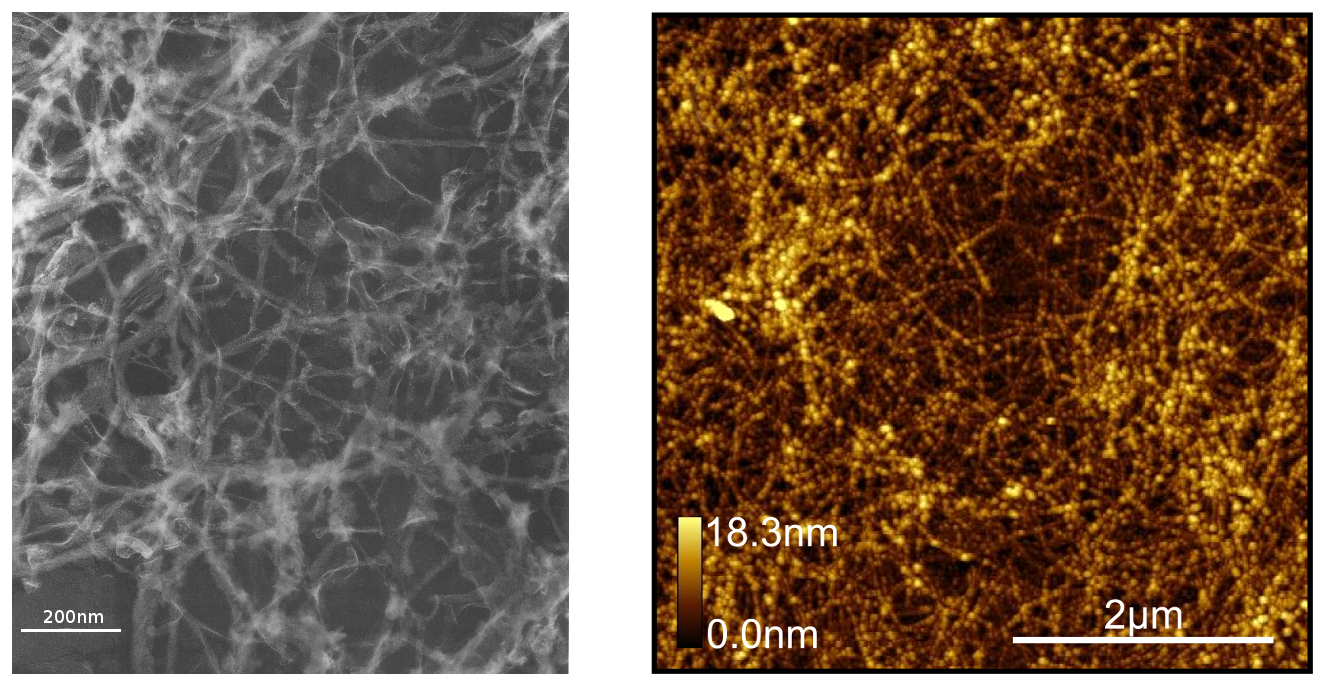
Macromolecular networks formed from tau and A-beta. Left: TEM image from Ruben [16] of intracellular neurofibrillary tangle composed of tau protein, extracted from human brain tissue. The sample was replicated by coating with 2 nm of platinum-carbon and 12 nm of carbon. The organic material was then dissolved in sulfuric acid and the metallic replica imaged by TEM. The NFT is composed of helical fibers which form a three-dimensional network with the fibers merging at points of contact, indicating that they are bound together. The spaces between the fibers form pores of consistent size, generally less than 100 nm. These features are characteristic of an irreversible macromolecular network or IRMAN, a structure identified by Terech as one of the four types of gel-forming networks [8]. Right: AFM image from Moores [21] of human amyloid beta peptide (A-beta), the primary constituent of extracellular plaques in Alzheimer's disease, induced to aggregate on a substrate. The image demonstrates that A-beta can also spontaneously assemble to form an amyloid fiber network with the structural characteristics of a gel.

Two different types of amyloid are seen in the brain in Alzheimer's disease, both of which are associated with degenerative changes. Tau protein, a normal component of the cytoskelton, aggregates inside neurons to form structures called neurofibrillary tangles (NFTs), while amyloid beta peptide (A-beta), a protein fragment, aggregates in the extracellular fluid (ECF) to form structures called plaques. Ruben [16] used transmission electron microscopy to image neurofibrillary tangles (NFTs) extracted from brain tissue and noted that the NFT resembles a gel in structure. His image (Figure 6, Left) shows that an NFT is a true molecular network rather than simply a convoluted fiber as the name might suggest. Tau demonstrates the helical structure commonly seen in gel-forming fibers, and, as in lysozyme, the fibers often appear to merge at points of contact. Moores [21] imaged A-beta amyloid fiber networks by AFM (Figure 6, Right); A-beta also demonstrates a three-dimensional network with pore size similar to that seen with lysozyme and tau. This network was formed by deposition of A-beta from solution directly onto a substrate. Extracellular plaques in the brain are likely formed by a similar process since free colloids in suspension would be carried out of the brain by the flow of ECF.

Gel formation has been reported with a wide variety of amyloid fibers, including insulin [22], 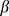-lactoglobulin [20], [23], and short synthetic peptides [24]. Protein aggregation under physiologic conditions may be very slow, however neurons are exceptionally long-lived and there is ample time for intracellular amyloid fibers to form and assemble into macromolecular networks. While the different imaging methods used limit our ability to make direct quantitative comparisons, both tau and A-beta amyloids appear to demonstrate the critical characteristics of an irreversible macromolecular network or IRMAN, one of the four principal types of gels identified by Terech [8]. The structure of lysozyme amyloid fiber networks appears similar in scale and geometry to amyloid networks formed from tau or A-beta. Further study is needed to compare the structure and properties of lysozyme gels with NFTs and plaques, however we suggest that lyzozyme can be a useful model for studying amyloid gel formation in disease.

### Effects on Biological Transport Mechanisms

Movement of a solute or suspended colloid in a gel is complex, and numerous models, many semi-empirical, have been proposed [25]. Mechanisms by which transport may be hindered include chemical bonding, colloidal interactions between the solute and fibers such as coulomb forces, steric hinderance to diffusion, mechanical blockage of particles close to or larger than the gel pore size, tortuosity, and perhaps most importantly, hydrodynamic drag between the fiber network and the solvent, which blocks convection.

#### Diffusion

Cole [26] suggested that amyloid gels might disrupt cell processes through their effects on diffusion. Dissolved molecular solutes are much smaller than the pore size of amyloid gels, however the rate at which they diffuse through a fluid is reduced in the presence of either a gel or a suspended polymer, primarily by steric obstruction [27]. Hydrogen bonds exposed on the fiber surface also bind surrounding water molecules into an ordered structure, increasing the effective fiber diameter. For solutes transported by simple diffusion, the ratio between the coefficient of diffusivity of a solute in a stationary fluid and in a hydrogel was first described analytically by Lauffer [28] as:
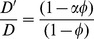



where:

D′ =  Coefficient of diffusivity in gel

D =  Coefficient of diffusivity in solvent


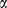
 =  shape constant for the obstructing structure, 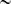
5/3 for randomly oriented rods


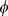
 =  volume fraction of the gel

The specific volume of lysozyme in water is reported to be.785 cm^3^/gm [29]. Thus, a typical amyloid gel produced with lysozyme 20 mg/ml will have a fiber volume fraction of no more than 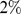, which would reduce the rate of diffusion of small molecules and ions by only 1.2%, an insignificant change. Similarly, Nicholson [30] reports minimal effects on small ion diffusion in a 2% gel.

Simple diffusion is not, however, the predominate mechanism of biological transport except at the very smallest scales. Diffusion is driven solely by concentration gradient, and the difference in concentration between source and destination required to maintain a uniform flux of a diffusant increases linearly with distance. For transport over significant distances the difference between the concentration at the source and the destination exceeds the tolerance of biological systems, thus transport often requires other mechanisms.

#### Convection

Convective flow does not depend on concentration gradient and is thus not limited by distance. In a gel, convective transport can occur whenever there is bulk flow of solvent through the fiber network. In hydrogels with pore size 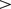
100 nm the transport of solutes is dominated by convection rather than diffusion [25]. Consistent with this, Tamagawa [31] found that transport of a dye in polyacrylamide gel was greatly accelerated if a small number of macropores (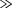
100 nm) were introduced. However these large pores were not thermodynamically stable and were rapidly filled in by the formation of new gel. We found pore size to be quite uniform in mature lysozyme gels, suggesting there are stabilization processes which fill in macropores but prevent fibers from aggregating into a solid mass. Pore sizes in most amyloid gels appears to be in the 50–80 nm transition range which permits diffusion but strongly impedes convective flow.

Forced convection is produced by a pressure difference between a source and destination; physiologic convection in the ECF has been measured at.6
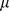
m/s [32]. When a gel is present hydrodynamic drag is greatly increased. Levick [33] examined a variety of tissues with gel-like matrices and demonstrated that the interstitial space in such tissues is filled by a network of fibers of two principal types, the first composed of glycosaminoglycan and the second of collagen. Hydraulic conductivity was found to range from 
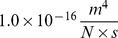
 for femoral head cartilage to 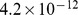 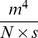 for vitreous humor. Some tissues contain cells which receive nutrients solely by convective flow through a gel, however this appears to occur only in tissues with a relatively low metabolic rate. Brain, in contrast, has one of the highest sustained metabolic rates of any tissue, consuming 20% of the body's total energy budget [34].

In the extracellular compartment of the brain, based on the distances involved, we would expect convection to be critical in the transport of nutrients from capillaries to the cell body, in the removal of metabolic wastes, and in the transport of neurotransmitters across synaptic spaces. The brain is uniquely devoid of lymphatic channels. Tracer experiments by Cserr[35] demonstrated that ECF is secreted from capillaries at a rate of .1–.3
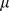
g per gm of brain tissue per minute, and flows through the tissue extracellular space to the ventricular and cortical surfaces where it enters the cerebrospinal fluid (CSF).

When particles are transported through a fluid by diffusion, their rate of movement varies inversely with hydrodynamic radius; small particles consistently diffuse more rapidly than larger ones. A similar effect is seen when particles of different sizes are transported by forced convection through a gel [36]. However Cserr found that the rate of movement of tracer molecules injected into brain ECF *in vivo* was uniform across a wide range of molecular sizes. This confirms that dissolved molecules do not simply diffuse through brain tissue but are transported by convection, i.e. bulk flow of the ECF, and that the channels through which this flow occurs are not normally obstructed by gel-forming fiber networks.

If an interstitial channel becomes filled with amyloid gel, how would this affect flow of the ECF? Flow in a channel can be characterized as laminar or turbulent based on Reynolds number:
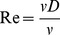



In a typical ECF channel in the brain we may assume, as a first approximation:


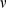
 =  kinematic viscosity  = 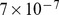
m^2^/s


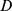
 =  hydraulic diameter  = 10^−6^ m


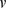
 =  mean velocity of fluid  = 10^−7^ m/s

Substitution of the above values gives Reynolds number 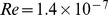 which confirms that flow is laminar. For laminar flow through an unobstructed channel, volumetric flow rate is given analytically by the Hagen-Poiseuille equation:
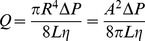



where:


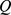
 =  volumetric flow rate


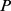
 =  difference in pressure over the channel length


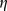
 =  dynamic viscosity


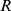
 =  channel radius


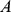
 =  channel cross sectional area


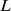
 =  channel length

If the channel becomes filled with a porous medium such as amyloid gel, according to Darcy's Law[37],
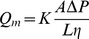



where:


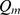
 =  volumetric flow rate through a medium


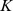
 =  Darcy permeability coefficient


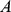
 =  cross-sectional area of gel (perpendicular to flow)


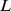
 =  length of gel in direction of flow


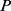
 =  pressure difference across the gel

The Darcy permiability coefficient 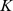 can be approximated under the Carman-Kozeny model of permeability [38] as:
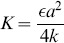



where:
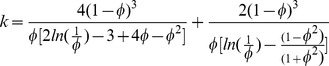



and:


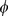
 =  volume fraction of network fibers


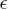
 =  porosity  = 1-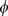



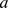
 =  pore size


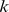
 =  shape factor

For a typical amyloid gel with a volume fraction of.02 and pore size of 80 nm, 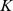 = 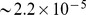 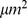. If an unobstructed channel becomes filled with amyloid gel, while pressure difference along the channel remains unchanged, flow will decrease by
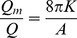
where 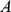 =  channel area in 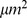. The effects of blockage by gel increase with channel size, but even for a relatively small channel with an area of 1 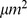:



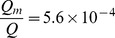



Thus convective flow will be effectively halted if a channel as small as 1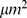 in cross-sectional area becomes blocked by amyloid gel over even a fraction of its length. The absence of convection effectively halts transport of both large and small solutes, although steric hindrance and filtration effects produce additional reductions in transport for larger structures.

If an interstitial channel becomes partially blocked at one point by amyloid gel, the effective channel cross-section will be reduced at the obstruction and local average flow velocity will be increased, as will fluid shear at the channel walls. Because amyloid deposition is accelerated by increased fluid shear [6] we would expect amyloid to preferentially form at the point where the flow is constricted by the obstruction, until the blockage is complete.

As the hydraulic permeability of the extracellular space decreases, the rate of ECF flow would be expected to also decrease if the pressure gradient between the capillaries and the CSF remains constant. This may contribute to the reduced CSF production seen in Alzheimer's disease. In contrast, CSF production is unaffected in Parkinson's disease, in which amyloid fiber formation is limited to the intracellular space [39]. Reduced secretion and convective flow of ECF may also reduce the flow of nutrients from capillaries to neurons, and of waste products from the ECF to the venous system. Finally, the emptying of neurotransmitter vesicles across the plasma membrane into the ECF and movement of neurotransmitters across the synapse requires convective flow, which could be hindered by the presence of extracellular gels formed from A-beta fibers in the synaptic cleft.

Measurement of fluid convection in the brain at milimeter resolution is possible *in vivo* using diffusion tensor imaging. Using this method Head et al.[40] reported increased mean diffusion in some brain regions in Alzheimer's disease. However this study examined directional flow along major nerve tracts and may reflect gross anatomical changes as large numbers of neurons are lost in late disease, rather than local ECF flow in the region of neuron cell bodies early in the disease.

#### Movement of Intracellular Transport Vesicles

A fiber network can block the movement of structures larger than its pore size by simple filtration. Even the normal cytoskeleton prevents movement of mitochondria and similar-sized structures into certain parts of the cell, apparently because of the spacing and orientation of the microtubules [41].

Axoplasmic flow in neurons is bidirectional and therefore cannot be due to convection of cytoplasm alone. It requires both anterograde and retrograde movement of colloidal-sized vesicles by active transport along microtubules of the cytoskeleton [42]. These vesicles are typically larger than the pore size of gels and their movement would be blocked if the microtubule passes through an amyloid fiber network. Tau NFTs can clearly be larger than 1
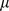
m *in vivo* [16]. Gel particles of this size within an axon or dendrite could block all axoplasmic flow in the process. Consistent with this, loss of neuronal processes is often seen prior to cell death in neurodegenerative disease [43], [44].

Neurotransmitters are transported intracellularly in vesicles which are 40–100 nm in diameter [45], [46]. Movement of such vesicles through a gel would be impeded by collisions with fibers, and because of variability in pore size vesicles would at some point encounter pores smaller than their diameter and be blocked. This could limit availability of neurotransmitters at the synapse. If trapped vesicles ultimately break down, neurotransmitters may be released into the cytoplasm; the presence of free neurotransmitter metabolites has been proposed as a promoter of further amyloid aggregation [47].

### Effects of the Solution Environment

Amyloid fiber formation, the assembly of fibers into molecular networks, and the conversion of fluids into gels all involve colloidal interactions. The kinetics of these processes are highly dependent upon environmental conditions including temperature, pH, and the nature and concentration of counterions and amphoteric molecules that can shield or enhance coulomb repulsion or alter molecular adhesion. Understanding these effects may allow us to inhibit colloidal aggregation of proteins by manipulating the environment within and around the neurons in the brain. A comprehensive predictive theory of amyloid aggregation is needed if we are to identify optimal therapeutic strategies for amyloid disease. One step toward such a theory may be the development of a multidimensional phase diagram showing the relationship between amyloid aggregation and each of the environmental parameters that affect its kinetics or pathways.

## Conclusion

In our study, increasing salt concentration did not affect fiber formation, but inhibited the transition of amyloid fibers to a helical conformation and accelerated the self-assembly of fibers into macromolecular networks. Such networks convert the surrounding fluid into a gel. The effects of amyloid gels on simple diffusion of solutes are modest. However active transport of colloidal-sized vesicles is critical to the function of neuronal processes; amyloid gels can block this process by filtration. Convection, or bulk flow, is essential to transport of dissolved molecules in the extracellular fluid; amyloid gels can halt convection by creating hydrodynamic drag. The primary cause of cell death in amyloid neurodegenerative disease may not be chemical toxicity, but rather the physical effects of amyloid gels on the movement of fluids and suspended particles.
