# Correction: Gel Formation in Protein Amyloid Aggregation: A Physical Mechanism for Cytotoxicity

**DOI:** 10.1371/journal.pone.0104152

**Published:** 2014-07-25

**Authors:** The PLOS ONE Staff

The fifth author’s name and affiliation are incorrect. The correct name is Lisa Cole Burnett. The correct citation is Woodard D, Bell D, Tipton D, Durrance S, Burnett LC, et al. (2014) Gel Formation in Protein Amyloid Aggregation: A Physical Mechanism for Cytotoxicity. PLoS ONE 9(4): e94789. doi:10.1371/journal.pone.0094789. The correct affiliation for Lisa Cole Burnett is Department of Chemical Engineering, Florida Institute of Technology, Melbourne, Florida, United States of America.
